# A review on recent advances in mass spectrometry analysis of harmful contaminants in food

**DOI:** 10.3389/fnut.2023.1244459

**Published:** 2023-08-01

**Authors:** Qiannan Sun, Yide Dong, Xin Wen, Xu Zhang, Shijiao Hou, Wuduo Zhao, Dan Yin

**Affiliations:** ^1^College of Chemistry, Zhengzhou University, Zhengzhou, Henan, China; ^2^Food Laboratory of Zhongyuan, Zhengzhou University, Zhengzhou, Henan, China; ^3^School of Ecology and Environment, Zhengzhou University, Zhengzhou, Henan, China; ^4^Center of Advanced Analysis and Gene Sequencing, Zhengzhou University, Zhengzhou, Henan, China

**Keywords:** mass spectrometry, food analysis, mycotoxin, pesticide and veterinary drug residues, acrylamide, food additives, polychlorinated biphenyls and dioxins

## Abstract

Food safety is a widespread global concern with the emergence of foodborne diseases. Thus, establishing accurate and sensitive detection methods of harmful contaminants in different food matrices is essential to address and prevent the associated health risks. Among various analytical tools, mass spectrometry (MS) can quantify multiple impurities simultaneously due to high resolution and accuracy and can achieve non-target profiling of unknown pollutants in food. Therefore, MS has been widely used for determination of hazardous contaminants [e.g., mycotoxin, pesticide and veterinary drug residues, polychlorinated biphenyls (PCBs), dioxins, acrylamide, perfluorinated compounds (PFCs) and p-Phenylenediamine compounds (PPDs) in food samples]. This work summarizes MS applications in detecting harmful contaminants in food matrices, discusses advantages of MS for food safety study, and provides a perspective on future directions of MS development in food research. With the persistent occurrence of novel contaminants, MS will play a more and more critical role in food analysis.

## Introduction

Over the past few years, food safety has become a growing global concern. The food safety analysis included the daily presence of various contaminants and residues, such as plant protection products, veterinary medicines, mycotoxins, chemical impurities, additives, packaging materials, and impurities added during processing packaging materials (1). PFCs, polybrominated diphenyl ethers (PBDEs), dioxins, mycotoxins, and PCBs, are common food contaminants that pose risks to human health. Therefore, identifying these contaminants is critical. Maximum residue limits (MRLs) have been stipulated in many countries to limit the use of contaminants in food to minimize public health concerns (2). Generally, the quantification of unknown pollutants in food has been determined by conventional methods, such as gas chromatography (GC) and high-performance liquid chromatography (HPLC). However, these approaches have several limitations in determining multiple residues simultaneously. More effective and powerful tools are needed due to the complexity of food samples.

MS has been widely applied in protein identification, biological analysis, and food analysis. It can be used in different scanning modes, including Full Scan, Daughter Ion, Parent Ion, Neutral Loss, and multiple reaction monitoring (MRM). Moreover, volatile and non-volatile components, high and low ionic components, high and low melting point substances, and their combinations without ionizable groups are analyzed by MS-based techniques (2). MS-based detectors offer excellent recovery, selection, sensitivity, reproducibility, and minimum interference. MS is widely used to analyze diverse food residues, such as vegetables (3), fruits (4), meat (5). MS combines powerful chromatographic separation to identify and confirm the presence of target compounds. It is a futuristic approach with a wide range of applications like, identifying separated compounds, elucidating the structure of compounds, and investigating degradation pathways.

Especially high resolution mass spectrometry (HRMS), it offers the advantage of higher sensitivity and full scanning. It provides a better understanding of sample composition than LC–MS/MS. HRMS has been described as a modern tool to test drug residues. Large-scale analytical screening methods have been published for veterinary medicines analysis in different food and animal matrices (6). HRMS is the most promising tool for the development of non-targeted methods. Besides, due to its excellent sensitivity and specificity, GC–MS has evolved as an important technique in recent years for food contaminants and is widely used for low molecular weight compound identification (7). According to the survey, LC–MS/MS is known for its ability to develop a wide range of residues and a large number of methods. It is considered the most popular and well-established analytical technique, but has shortcomings in some aspects such as limited number of analytes, inability to screen for unknowns and time-consuming assay setup.

The purpose of this paper is to [1] highlight the advantages of MS in detecting hazardous contaminants in food, [2] summarize recent advances in MS methods for mycotoxins, pesticide and veterinary drug residues, acrylamide, food additives, PCBs, dioxins, PFCs, and PPDs analysis over the past 10 years, and [3] overview the future applications of MS methods in food research.

### Mycotoxins

Mycotoxins, a common food contaminant, are poisonous secondary metabolites produced by fungi, such as Aspergillus, Penicillium, and Fusarium. Their presence in hot processed foods is a worldwide concern. These naturally occurring compounds are very toxic to humans and enter the human body via the food web, causing hepatotoxicity, genotoxicity, immunosuppression, nephrotoxicity, teratogenicity, and carcinogenicity (8). Mycotoxins are found in many agricultural products, such as grains and nuts. Therefore, considerable attention has been paid to mycotoxins analysis. Table 1 summarizes the use of MS in analyzing natural mycotoxin in foods.

**Table 1:** Applications of MS for analysis of mycotoxin in foodstuffs.

Foodstuffs	MS	Analytes	Sample preparation	LODs	Reference	
Nuts	UHPLC–MS/MS	AFB1, AFB2, AFG1, AFG2 and OTA	Grinding and sieving	–	(9)	
Cereals and cereal-based food	LC–MS	Mycotoxins	–	–	(10)	
Raisin, pistachio, peanut, wheat flour, spice, and chili samples	LC–MS/MS	AFB1, AFB2, AFG1, AFG2, and OTA	Grinding and homogenization	–	(11)	
Bee products	HPLC, LC–MS/MS	DON, HT2, T2, OTA	Drying	0.0004–0.012 ng/mL	(12)	
Table-ready foods	LC–MS/MS	Aflatoxins B1, B2, G1, and G2, fumonisins B1 and B2, ochratoxin A, and zearalenone	Homogenisation	0.01–2.4 μg /kg	(13)	
Malted barley and beer	QuEChERS-LC-QTOF-MS	AFB1, AFB2, AFG1, AFG2, BEA, ENA, ENA1, ENB, ENB1, FB1, HT-2 toxin, T-2 toxin, OTA, MON, DON, ZEA and STG	–	0.01–15 μg/kg	(14)	
Soy-based burgers	UHPLC-Q-Orbitrap HRMS	DON, 3-ADON, 15-ADON, DAS, HT-2, T-2, ZON, OTA, AFM1, AFB1, AFB2, AFG1, AFB2, FB1, FB2, ENNA, ENNA1, ENNB, ENNB1, AOH, AME, and 12 isoflavones	–	21 mycotoxins: 0.05–0.50 μg/kg 12 isoflavones: 0.01–0.10 μg/kg	(15)	
Wheat, rye, and maize flour	DART-Orbitrap MS	Fusarium spp., Claviceps Spp, Aspergillus spp., Alternaria spp	–	25–250 μg/kg	(16)	

–, not mentioned.

For more accurate quantification of mycotoxins, LC–MS is now the most widely used technique for the detection of mycotoxins in food (17), especially for analyzing grain and grain products (10). Similarly, GC–MS also plays a role in mycotoxin screening and quantitative analysis (18). Singh et al. (19) summarized the mycotoxin analysis using traditional (HPLC) and advanced methods (LC–MS and GC–MS) and analyzed mycotoxins characteristics. The result showed that LC–MS and GC–MS have better sensitivity than conventional methods. Another work discussed mycotoxins in cereal products and summarized the most commonly used detection techniques, including LC-electron spray ionization (ESI)-MS. Sample pre-treatment was carried out using the minimal clean-up method, QuEChERS, which is fast, simple, inexpensive, effective, durable and safe. In this work, the ranking of cereal products according to the number of trials was cereals > cornflakes > bread > breakfast > flour > baby products > pasta > other products, suggesting that research on mycotoxins in cereal products had attracted much attention (20).

Studies have reported that 120 food matrices have been analyzed for mycotoxins using LC–MS/MS technique (11). Alsharif et al. (11) used LC–MS/MS to determine mycotoxins in raisins, pistachios, peanuts, wheat flour, spices, and pepper samples simultaneously. Sample pretreatment was performed by the QuEChERS technique, with recoveries ranging from 81.94 to 101.67%. Similarly, Lee et al. (13) used LC–MS/MS and stable isotope dilution to analyze multiple mycotoxins in ready-to-eat foods, yielding good results. Mycotoxins are also present in bee products，which can boost immunity and are important diets for adults and children. However, the health effects of mycotoxins-contaminated bee products in healthy individuals are unknown. Keskin et al. (12) performed HPLC-UV analysis of mycotoxins, followed by LC–MS/MS, to detect the positive samples and improve sensitivity. It was found that mycotoxins consumed from certain amounts of bee products do not pose a health risk. Beans are highly nutritious foods, and their consumption is becoming increasingly popular. However, mycotoxins are found in beans. A work by Acuña-Gutiérrez et al. (21) summarized the mycotoxins detection techniques in beans, including GC–MS/MS and HPLC–MS/MS. Compared to GC–MS/MS and HPLC–MS/MS, HRMS is also used for the detection of mycotoxins with higher sensitivity and efficiency. Rodríguez-Carrasco et al. (15) first used acetonitrile-based extraction and ultra-high-performance liquid chromatography coupled with quadrupole exactive orbitrap high resolution mass spectrometry (UHPLC-Q-Exactive-Orbitrap HRMS) to analyze mycotoxins in soy burgers with recoveries in the range of 78 to 108% and precision less than 12%. The limits of quantitation (LOQs) of all investigated compounds were in the low ng/g range, and the co-existence of mycotoxins was observed in approximately all samples. Similarly, a technique to detect 19 mycotoxins in grain flour matrices using real-time orbitrap mass spectrometry (DART-Orbitrap MS) was developed and validated by Tsagkaris et al. (16), based on a LC method combined with both a hybrid HRMS (q-Orbitrap MS) and a low-level resolution mass spectrometry (triple quadrupole, QqQ) detectors. Compared to the liquid–liquid method, the DART-Orbitrap MS exhibited high throughput, speed and excellent detection capability, monitoring up to 96 samples in a single runtime of approximately 40 s, which is crucial for the regulation of mycotoxins. The emerging DART-Orbitrap MS can improve and accelerate the detection of selected mycotoxins in cereal samples compared to LC–MS, and the superiority of this technique makes it more promising for future applications in the detection of mycotoxins in a wide range of foods. For mycotoxin analysis in fatty matrices such as nuts, Castilla-Fernández et al. (9) isolated mycotoxins from walnuts by solid–liquid extraction (SLE) and then analyzed them using UHPLC–MS/MS. Due to the strong matrix effect in fatty matrices, the dilution-injection method was the newest trend and solved the problem of the matrix effect. This method has the advantages of simplicity, minimal loss of analytes, high sample flow rate, and many analytical classes covered. Six purification columns were used to purify fatty matrices, none of which effectively reduced the strong matrix effects encountered by SLE. Finally, weak or negligible matrix effects were obtained by applying 1:100 dilutions of SLE extracts. LOQs met The European Union (EU) requirements, and satisfactory recovery and precision were obtained. To reduce the effects of matrix effects, Lago et al. (14) first evaluated a liquid chromatography coupled with quadrupole time-of-flight mass spectrometry (LC-QTOF-MS) for concurrently analyzing legal and newly discovered mycotoxins in malt and beer. The method overcame matrix effects and is rapid, requiring only 1.2 min for simultaneous analysis of emerging mycotoxins. The MS was applied to detect mycotoxins using MRM and total mode, with satisfactory linearity and recoveries. In summary, the detection of contaminants in food samples with complex matrices and difficult sample processing has been a challenging task. The development of LC-QTOF-MS with low matrix effects and high speed has to accelerated the study of food contaminants.

### Pesticides residues

Pesticides can enter the food chain during their application. Food is common exposure to pesticide at low doses. Surprisingly, some substances become more potent when they accumulate in the food chain. When the concentration reaches a critical level, damage to human health becomes more serious (22). Pesticides mainly have higher risks to the nervous system (23). Also, multiple sclerosis, cancer, and several chronic diseases have been linked to long-term pesticide exposure. Table 2 summarizes how MS analyzes residues in pesticides residues.

**Table 2:** Applications of MS for analysis of pesticides residues in foodstuffs.

Foodstuffs	MS	Analytes	Sample preparation	LODs	Reference
Vegetable and fruit	GC–MS/MS and UHPLC-QTOF-MS	52 Pesticides	Homogenisation, Freezing at −20°C	–	(32)
Beef, pork, and chicken	GC–MS/MS	Pesticides	Homogenisation, Freezing at −20°C	–	(5)
Sweet pepper	LC–MS/MS and GC–MS/MS cross-checking analysis	Pesticide	Grinding and homogenization	LC–MS/MS: 0.03–0.5 μg/kg, GC–MS/MS: 0.9–2.0 μg/kg	(40)
Pigeonpea grains	GC–MS/MS and LC–MS/MS	79 Pesticides	Homogenisation	0.53–3.97 μg/kg	(41)
Passion fruit	LC–MS/MS and GC–MS/MS	Pesticide	Homogenisation	–	(42)
Pepper, chili peppers and its sauce product	LC–MS/MS and GC–MS/MS	47 Pesticides	Homogenisation	–	(38)
Chinese cabbage	Modified QuEChERS-UPLC–MS/MS	8 Neonicotinoid insecticides	Homogenisation, Freezing at −20°C	–	(3)
Apple, cottonseed, pepper, tomato, grape and pea	Modified QuEChERS-UPLC–MS/MS	Xinjunan pesticide residue	Homogenisation	–	(31)
Fish and shrimp	QuEChERS-(LC-MS/MS)	Multi-pesticide residues	Homogenisation, Freezing at −20°C	0.1–3 μg/kg	(35)
Twelve common vegetables and fruits (cherry, pumpkin, eggplant, potato, pear, grape, peanut, cucumber, spinach, wolfberry, wheat, and sugarcane)	QuEChERS-HPLC-MS/MS	Cyflufenamid	Homogenisation, Freezing at −20°C	0.2–0.4 μg/kg	(30)
Foods of plant originpeaches (grapes, brown rice and soybeans)	Derivatization-QueChERS-HPLC-MS/MS	Zinc-thiazole	Grinding and homogenization	0.001 mg/L	(34)
Chilli and Sichuan pepper	LC-Q-TOF-MS	Pesticide	–	0.6–1.7 μg/kg	(39)
Baby foods	QuEChERS-UHPLC-Q-Orbitrap MS	21 Pesticides and 4 aflatoxins	–	0.02–4 μg/kg	(36)
Spices black pepper, cardamom, chili, coriander, cumin, and turmeric	UHPLC-Q-Orbitrap MS	Pesticides	Homogenisation	-	(33)
Baby foods	UVALLME-GC-ITMS	19 Organophosphorus pesticides (OPs)	–	0.2–1.3 ng/g	(23)
Tomato and bell pepper	TD-ESI-MS/MS	Pesticides	Homogenisation	-	(43)
Tea samples	UA-MSPE-UHPLC–MS/MS	Metalaxyl, napropamide and epoxiconazol	Grinding storing at 25°C	0.02–0.05 ng/ g	(37)

–, not mentioned.

Pesticides can protect crops and avoid pests and diseases. However, there are many abuse instances of pesticides due to their widespread use, leading to serious pesticide residues in vegetables, fruits, water (24), and soil (25). Due to their wide variety, analyzing pesticides in food is daunting since the matrix concentration in foodstuffs is higher than that of pesticides. Pesticide contamination also occurs and is increasing in food of livestock origin, bringing comprehensive multi-residual analysis of plant protection products to the global attention forefront. EU has now set MRLs for various pesticides in different foods to monitor risks to people’s health and the natural environment (26). MS is highly sensitive and can unambiguously detect trace amounts of samples, and it is now becoming increasingly common for detecting pesticide residues, particularly in food (27). Different MS-based techniques have been frequently used to detect food contaminants, such as HPLC-MS, GC–MS, ultra performance liquid chromatography–tandem mass spectrometry (UPLC-MS/MS) and GC–MS/MS (28), UHPLC–MS/MS, UHPLC-QTOF-MS, DART-MS (29), Desorption electrospray ionization mass spectrometry (DESI-MS).

**Figure 1:**
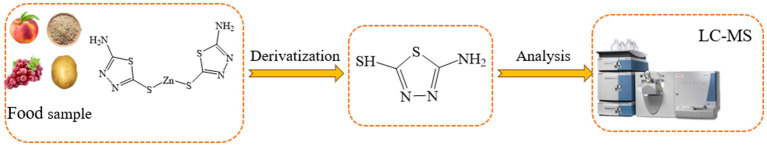
Improved derivatization-QuEChERS method for HPLC–MS/MS analysis of fungicide residues in foods of plant origin (34).

QuEChERS is frequently used for sample pretreatment with the advantage of quick, easy, affordable, efficient, reliable, and secure. Liu et al. (30) reported the improved QuEChERS extraction technology for sample preparation. Acetylene was used as the extraction agent, and SAX, XFM42, and C18 as the stationary phase. The proposed HPLC-MS/MS was used to identify and quantify fipronilide in food samples and the LOQs range was 0.5 to 50 μg/kg. Han et al. (31) pretreated food samples using a modified QuEChERS method, followed by UPLC-MS/MS with simultaneous MRM concerning quantification *via* a standard outer method. The mean recoveries of the developed were 75.6–106.2%, with an RSD of ≤8.8%. Kottadiyil et al. (32) adapted the QuEChERS method to detect and quantify pesticide residues in vegetables and fruits by GC–MS/MS and UHPLC-QTOF-MS and used MRM mode to quantify samples. This model exhibited high specificity, selectivity, and sensitivity in SCAN and SIM (single ion monitoring) modes. Goon et al. (33) employed a new method to screen non-targeted pesticides multi-residues in spice matrices by UHPLC-Q-Orbitrap MS. Samples were extracted using the standard QuEChERS method. Compared with LC–MS/MS, recoveries obtained by the UHPLC-Q-Orbitrap MS method were in the 78–100% range with successive full instrument scans and stable quantitative performance. In addition, HLB purified samples, and the recoveries ranged from 70 to 120% with an accuracy RSD of <20%. Chen et al. (34) used a developed derivatization-based QuEChERS approach for online HPLC–MS/MS detection of fungicide pesticide residues in vegetable-based food products (Figure 1). Good performance can be obtained in the range of 0.001–1 mg/L under the negative ionization scan mode. Dispersive solid-phase extraction (DSPE) is a new sample pre-treatment technique that is easy, fast, efficient, accurate and sensitive. Shin et al. (35) used d-SPE for sample pre-processing and cleaning by octadecylsilane (C18) and primary secondary amine (PSA) as d-SPE absorbents. Multiple agrochemicals in fish and shrimps were analyzed simultaneously by LC-MS/MS with good recoveries (70–125%). Similarly, Prata et al. (36) used QuEChERS extraction and d-SPE purification method to monitor pesticides and aflatoxins in infant food. Quantifying multiple contaminants and aflatoxin levels in infant formula sold in Brazil by UHPLC-Q-Orbitrap MS technique achieved low LODs and LOQs.

Multi-walled carbon nanotubes are often modified as substrates for the extraction and adsorption of contaminants. Synthetic magnetic amino-modified multi-walled carbon nanotubes (m-MWCNTs-NH_2_) were used for sample purification and enrichment of pesticides in tea samples (37). They used UHPLC–MS/MS and ultra-sound-based magnetic solid-phase extraction (UA-MSPE) to simultaneously determine medomel, diquat, and oxytetracycline in tea samples. UA-MSPE provided a simple, rapid, and efficient analysis for targeted samples with promising LODs and LOQs. In addition, three plant protection products were detected in different tea samples with a recovery higher than 75.1%, suggesting that the method can be used in assessing plant protection products in other matrices. Magnetic multi-walled carbon nanotubes (Fe_3_O_4_MWCNTs) were used as sorbents to detect mycotoxins and pesticides in cereals by QuEChERS extraction.

Pesticide residues are also present in Chili peppers and Sichuan peppercorns, which are consumed globally for their unique flavour and high nutritional value. In addition, the converted product is in high demand as an essential spice. However, to get high yields, unscrupulous traders use illegal pesticides. Studies have shown that LC–MS/MS methods can be applied to analyze spices’ pesticide residues; however, only target compounds can be identified. Using these methods for non-targeted screening is impossible (38). Liu et al. (39) established a combined LC-Q-TOF-MS-based technique that analyzed all relevant pesticides in peppers and chilies with LOQ ≤ 5 μg kg-1. Song et al. (38) developed a novel, single-step technique with quick and easy, requiring no further vortexing or washing steps. A small column was placed in an extraction spinner to remove unwanted material. The absorbents were MWCNT and PSA blended with salts. 47 typical pesticides in pepper, chili, and chili sauce were detected by LC–MS/MS and GC–MS/MS, with satisfactory recoveries. GC–MS/MS was used for volatiles and lipophilic compounds, while LC–MS/MS analyzed pesticides containing polar moieties which are not heat resistant and non-volatile. To improve the accuracy of the analysis of various pesticide residues, an analytical approach using both LC–MS/MS and GC–MS/MS was reported by Lee et al. (40) The data indicated that recoveries were between 70 and 120%, and cross-checking was more effective and reliable than a single method in identifying several pesticides. The LODs for LC–MS/MS were 0.03 to 0.5 μg/kg, and LOQs were 0.6 to 1.5 μg/kg. For GC–MS/MS, the LODs and LOQs were 0.9 to 2.0 μg/kg and 3.0 to 5.7 μg/kg, respectively. Harischandra et al. (41) developed the concurrent measurements for 79 different pesticide residues in wood beans using LC–MS/MS and GC–MS/MS with recoveries ranging from 70 to 120%. According to MRLs for wood beans, satisfactory precision (RSD < 20%) was achieved for detecting and quantifying pesticide residues under 10 μg/kg. Similarly, Mozzaquatro et al. (42) determined 80 pesticides, including 5 metabolites in passion fruit by LC–MS/MS and GC–MS/MS in a cross-synchronous analysis, allowing a more comprehensive and detailed sample determination. Therefore, the scattered omnidirectional solvent extraction method was adopted, and the results showed that the sample recovery was more than 70%; RSD ≤ 20%.

Ambient ionisation mass spectrometry (AMS) is a state-of-the-art technique that allows rapid chemical analysis without the need for sample preparation and chromatographic separation. It is now being used to detect food contaminants, particularly in the detection of pesticide residues on fruits and vegetables. Cheng et al. (43) developed a combination of multi-probe samples, TD-ESI, and composite MS/MS analysis methods to determine many trace pollutants in fruits and vegetables. The method involves desorption and ionization of samples collected on the probe in a TD-ESI source, followed by QqQ-MS detection in MRM mode. Samples collected by QuEChERS extraction combined with GC–MS/MS or LC–MS/MS showed similar results to those determined by TD-ESI-MS/MS, which is a simple and time-saving technique that can simultaneously monitor multiple MRM channels. Therefore, QuEChERS coupled with TD-ESI-MS/MS exhibits significant potential to quantify pesticide residues in agricultural products. Pesticide residues in food can cause human health risks, especially for infants and children. An ultrasonic-vortex-assisted liquid–liquid microextraction method coupled with gas chromatography-ion trap mass spectrometry (UVA LLME-GC-IT/MS) was proposed by Notardonato et al. (23). The method was quick, simple, inexpensive, and accurate for 19 organophosphorus pesticides (OPs) determination in baby food. The LODs and LOQs were in the 0.2–1.3 and 0.5–2.9 ng/g range, respectively. Based on the developed LLME extraction with subsequent GC-IT/MS analysis, the recovery was in the 81 to 109% range.

### Veterinary drug residues

Veterinary medicines can prevent, diagnose or treat animal diseases. Due to increased human demand for meat products, many people add veterinary drugs to livestock and aquaculture processes to achieve high yields. However, the overuse of veterinary medicines can contaminate the aquatic environment and affect the safety of drinking water. More importantly, some people use illegal veterinary medicines for their own benefit, which can pose a serious threat to human health and cause greater pollution of the environment. Therefore, the detection and quantification of these drugs in animal tissues cannot be ignored in terms of food safety, human health and the environment. Current techniques to detect veterinary drug residues in animal-origin food are GC–MS/MS, capillary electrophoresis-mass spectrometry (CE-MS/MS) (44), LC–MS/MS (45), hydrophilic interaction liquid chromatography–tandem mass spectrometry (HILIC-MS/MS), reversed-phase liquid chromatography coupled with tandem mass spectrometry (RPLC-MS/MS) (46), QTOF-MS (47), Quadrupole Mass Spectrometry (QMS), UHPLC–MS/MS (48) and HPLC-Q-Orbitrap HRMS. In this review, we summarized the application of MS in the analysis of veterinary drug residues, as shown in Table 3.

**Table 3:** Applications of MS for analysis of veterinary drugs residues in foodstuffs.

Foodstuffs	MS	Analytes	Sample preparation	LODs	Reference
Milk	UHPLC–MS/MS	25 Veterinary drugs	–	–	(48)
Milk	MSPE-(HPLC-MS/MS)	Veterinary drugs	Homogenisation	–	(49)
Infant formula	UHPLC–MS/MS	Veterinary drug	–	–	(50)
Livestock foods	UHPLC-QTRAP	155 Veterinary drugs	Homogenisation	0.5–5 μg/kg	(51)
Egg	HPLC-QTOF-MS	Veterinary drugs and pesticides	Homogenisation	–	(52)
Milk and fish tissue	UHPLC-QTOF-MS	143 Veterinary drugs and pharmaceuticals	Homogenisation	–	(47)
Food (dairy-meat, fish, egg, and animal by-products)	QuEChERS-LC-HRMS	Veterinary drug	Homogenisation	–	(53)
Animal source foods	UPLC-Q-Exactive Orbitrap MS	Veterinary drug	–	0.1–10 μg/kg	(54)
Milk, egg and meat	HILIC-MS/MS and RPLC–MS/MS	Veterinary drugs	Homogenisation	–	(46)
Beef	LC–MS/MS	Multi-class 115 veterinary drugs	Homogenisation Freezing at −20°C	–	(45)
Honey, pig muscle, cow milk, and chicken eggs	HPLC-HRMS/MS	81 Multiclass veterinary drugs	–	–	(55)
Beef and chicken	HPLC-Q-Orbitrap HRMS	146 Veterinary drugs	–	0.15–3.03 μg/kg	(56)
Livestock products, seafood, honey, and royal jelly	LC–MS/MS	Chloramphenicol	–	–	(57)
Milk	LC-MS/MS	VCM and NVCM	–	–	(58)
Milk	CE-ESI-MS/MS	Aminoglycosides (AGs)	–	0.10 μg/L	(44)

–, not mentioned.

Beef and dairy products are rich in nutrients and widely consumed by the public, but the residues of veterinary drugs in these should not be ignored. Jung et al. (45) used LC–MS/MS to identify and quantify 115 veterinary drugs and their metabolites in beef. The QuEChERS technique was developed by dispersive solid-phase extraction using EMR as a sorbent. Under the positive ion mode, only three veterinary drugs could not achieve the required recoveries, while the recoveries of the remaining 112 were 70.7 to 117.9%. Melekhin et al. (49) developed an HPLC-MS/MS-based method to determine multiple animal drug residues in milk quantitatively. Magnetic supercross-linked polystyrene (HCP/Fe_3_O_4_) was used to develop a MSPE without a deproteinization step. As a new form of SPE, MSPE can eliminate the limitations of traditional SPE, such as backpressure fouling, complex filtration, and solvent waste. In addition, MSPE has simple and rapid sample preparation, solvent savings, low cost, and high recoveries of analytes. This has been reported as the first and most complete MSPE method for multi-residue analysis of animal drugs in milk. Zhao et al. (50) used UHPLC–MS/MS to analyze approximately 150 veterinary drugs in baby formula milk and associated constituents. The method achieved a LOD of 1-10 ng/g in baby food, whole milk powder, and isolated milk proteins.

HRMS is now widely used in the field of detecting drug residues in food. LC-HRMS was used to extend the detection of veterinary drug residues in foodstuffs. The LC-HRMS-based technique applies to screening raw materials of animal origin (53). TOF/QTOF and Orbitrap are often combined with HRMS, TOF analyzers are particularly popular in analyzing drug residues. HPLC-QTOF-MS exhibits high resolution and mass accuracy, and its full scan acquisition offers large amounts of target and non-target identification data. Hou et al. (52) developed an HPLC-QTOF-MS multi-class screening approach for measuring veterinary medicines and pesticides in eggs. A one-time injection procedure was developed for the qualitative analysis. Similarly, veterinary drug and pharmaceuticals residue analysis in fish tissues and milk using UHPLC-QTOF-MS was reported by Dasenaki et al. (47) Detecting veterinary medicinal residues in beef and chicken samples is complicated due to their complexity. Wang et al. (56). used QuEChERS pretreatment and HPLC-Q-Orbitrap HRMS for quantitative screening. The obtained recoveries were 52.1 to 138.2%, and the RSDs were 0.4 to 17.7%. In addition, the LODs were 0.15–3.03 g/kg, and LOQs were 0.5–10 g/kg. The method improved the accuracy and range of screening in full mode. With the demand for faster and more accurate detection of food contaminants, this will also drive the development of HRMS in combination with more chromatographic techniques. However, scanning rates need to be improved and user-friendly data-processing software needs to be developed as the most promising analytical technique.

A high throughput SPE method was developed based on Oasis PRiME HLB. Wang et al. (51) used UHPLC-QTRAP for simple and efficient quantitative analysis of 155 veterinary drugs in cattle and chicken feeds in MRM-IDA-EPI positive and negative scan modes. The obtained LOQs were in the range of 0.5 μg/kg and 5 μg/kg, and LODs were between 2 μg/kg and 20 μg/kg. In addition, satisfactory recoveries were obtained for more than 17 of 20 analytes. Zhao et al. (54) used a UPLC-Q-Exactive-Orbitrap MS system to screen and analyze multiple foodstuff veterinary residues. The method was rapid, simple, and sensitive and consisted of two steps; extraction with 0.2% formic acid-acetonitrile water and purification with PRiME HLB SPE columns. The analyses were performed in positive and negative MS1/MS2 full scan modes, with targeted identification by full scan MS. Obtained detection limits were 0.1 to 10 μg/kg, and recovery was 79.2 and 118.5% in all matrices.

**Figure 2:**
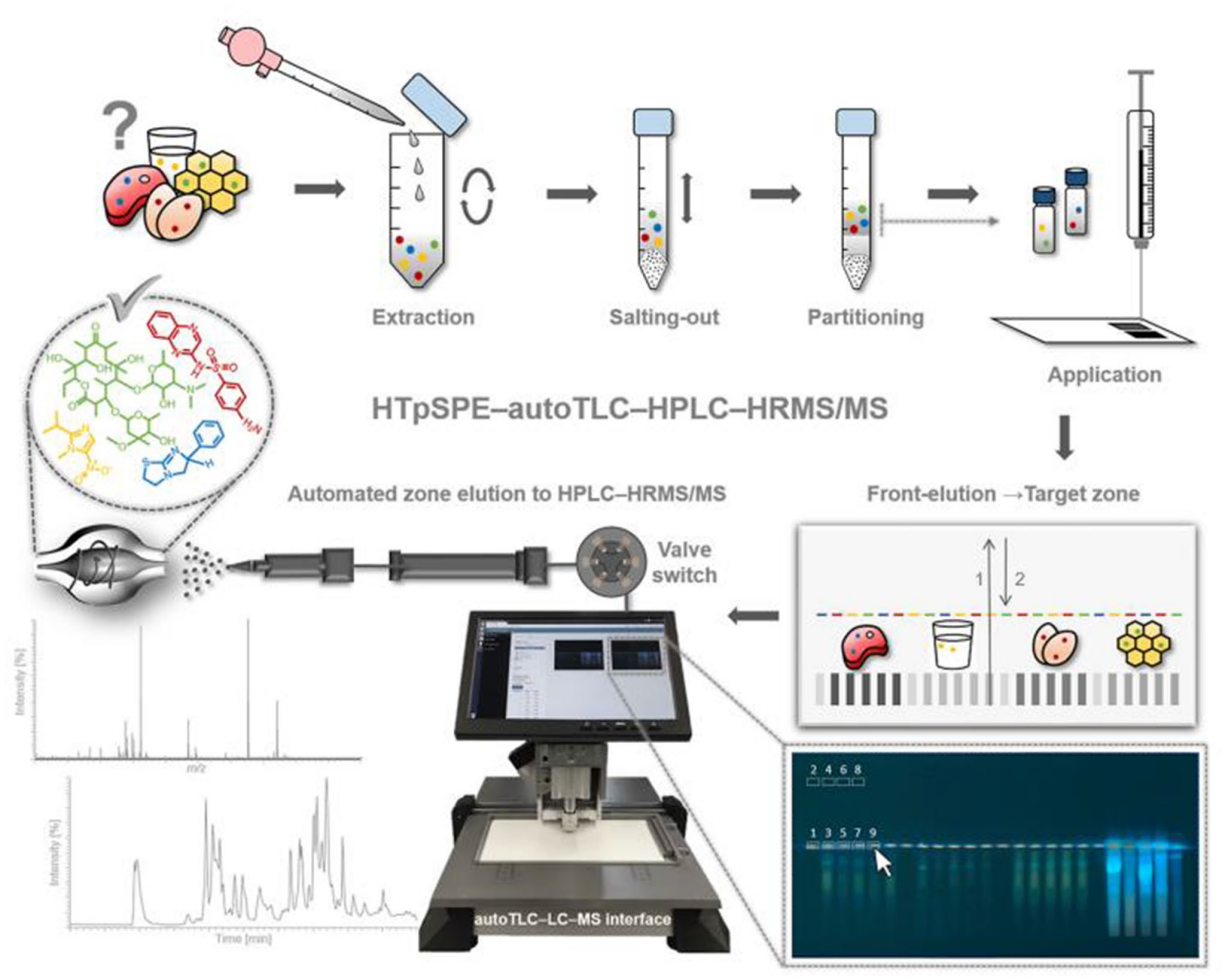
Workflow of the developed HTpSPE-UV/vis/FLD-autoTLC-LC-HRMS/MS method (55).

A 15-level multi-source method to screen hydrophilic and hydrophobic veterinary drugs in milk, eggs, and meat was developed by Chung et al. (46) Using HILIC-MS and HILIC/RPLC. The method typically achieved 70 to 120% recoveries with an RSD accuracy of <20%. Due to the limited screening capacity of current veterinary drugs, trace analysis is always time-consuming and expensive. Mehl et al. (55) first developed an automated HTpSPE-UV/vis/FLD-HPLC-HRMS/MS to screen 81 veterinary drug residues in honey, pig muscle, milk, and eggs (Figure 2). The developed method is green, fast, and solvent-saving compared to conventional methods. Based on automatic elution using an auto TLC-LC–MS interface, online HPLC separation, and detection by Orbitrap HRMS, most veterinary drugs were measured in honey at a 25 μg/kg concentration. The other three samples were found at a 5 μg/kg concentration.

Antibiotics are commonly used veterinary drugs. Currently, residues in meat products are one of the most severe food contamination problems, presenting a health risk to the public. Chloramphenicol (CAP), as an antibiotic, is used to treat diseases in honey bee larvae. However, CAP has high toxicity and less dose dependence in sensitive individuals. Therefore, it is banned in edible animals and honey bees. Kikuchi et al. (57) used LC–MS/MS to determine CAP and CAPG residues in animal products, fish and crustaceans, honey, and royal jelly. They used mixed hydrophilic–lipophilic copolymer for methanol extraction, beta-glucuronidase hydrolysis, and solvent exchange column purification. The method was simple and accurate, depicting excellent CAP and CAPG recoveries (79–109%). Recently, Zhou et al. (58) used LC–MS/MS to determine milk antibiotics (vancomycin and desmethyl vancomycin). They developed a surface molecularly imprinted solid phase extraction (SMISPE) based on a highly selective method. Sample handling and material effects were greatly reduced for targeting analytes, with recoveries in the range of 83.3–92.1%. Aminoglycosides (AGs) are antibiotics commonly found in milk and eggs, posing health risks to humans, including long-term exposure causing multiple allergic reactions. CE-ESI-MS/MS method was adopted by Yue et al. (44) to identify 4 AGs in milk simultaneously. CE-ESI-MS/MS combined with SPE and t-ITP showed recoveries above 76.20% for all AGs. Separation conditions were simpler than LC–MS, without ion-pairing reagents in the mobile phase.

In summary, LC–MS is a satisfactory technique for detecting pesticide and veterinary residues in food. Compared with other MS methods, LC–MS has the advantages of high sensitivity and selectivity, wide linear range and the ability to simultaneously analyze multiple pesticide and veterinary drug residues in food. This makes LC–MS become an important analytical tool in food safety monitoring and quality control. However, the high cost of LC–MS, insufficient resolving power, strong matrix effects and sometimes false-positive results pose challenges for the detection of pesticide and veterinary drug residues in food. Therefore, development of more superior MS detection methods is still needed.

### Acrylamide

Acrylamide is a regulated compound. It is used in food contact materials, plastics, paper, printing inks, etc. The human body can be exposed through a variety of routes, including the digestive tract, the respiratory tract and the mucous membranes of the skin. Food is the main source of acrylamide in humans. Acrylamide is produced during high-temperature processing, especially in high-protein and carbohydrate-containing foods, mostly found in processed food such as chips, crisps, bread, biscuits, crackers, and breakfast muesli. They are ingested with food and dispersed in the human body. Human exposure to acrylamide may result in toxicological effects (neurotoxicity, genotoxicity, carcinogenicity, reproductive toxicity and so on). Therefore, there is an urgent need to detect and quantify acrylamide in processed foods. Table 4 summarizes the use of MS for the analysis of acrylamide.

**Table 4:** Applications of MS for analysis of acrylamide in foodstuffs.

Foodstuffs	MS	Analytes	Sample preparation	LODs	Reference
Bread and bakery products	LC-ESI-MS/MS	Acrylamide	Homogenisation Freezing at −18°C	<10 μg/kg	(59)
Pork meatballs, beef meatballs, and chicken meatballs	UPLC-MS/MS	Acrylamide, 5-hydroxymethylfurfural, and 2-amino-1-methyl-6-phenylimidazo [4, 5-b] pyridine	Freeze-dried for 48 h	1.57 μg/L	(60)
Cricket flour, a cricket flour with chocolate, cricket bars and cricket crackers	HPLC-QqQ-MS/MS	Furanic compounds and acrylamide	Grinding, sieving and homogenising	–	(61)
Biscuit	LC-ESI-Orbitrap HRMS	Acrylamide	Grinding, sieving and homogenising	3.55 μg/kg	(62)
Potato chips, crackers, chanachur, biscuits, potato crisps, breakfast cereals, french fries, cake, bread	GC–MS/MS	Acrylamide	Homogenisation	6–12 μg/kg	(63)
Cookies, French fries, ground coffee, and brewed coffee	HRMS	Acrylamide	Homogenisation stored at −20°C	2.65 ppb	(64)

–, not mentioned.

LC–MS/MS and GC–MS/MS are the most commonly used methods to analyse acrylamide in food. Hasan et al. (63) used GC–MS/MS to determine acrylamide content in 180 hot-processed carbohydrate-rich foods. According to this analysis, the highest levels of acrylamide were found in crisps. Acrylamide has the characteristics of high polarity, low volatility and low molecular weight. Therefore, derivatisation is required for the determination of acrylamide by GC–MS/MS. LC–MS/MS is relatively easy to perform and is now the gold standard for accurate quantification of acrylamide in food (65). Mousavi Khaneghah et al. (66) investigated acrylamide concentrations in different kinds of food. They summarized the acrylamide concentrations in relevant foods in daily life, such as potato-based foods > fried foods > breakfast cereals > coffee > chocolate > baby food > bread > cookies > desserts > cakes > cereals > nuts. Similarly, Andačić et al. (59) reported that bakery products contributed approximately 37.2% to overall acrylamide exposure in Croatia. QuEChERS extraction of acrylamide, purification with PSA, and quantification with LC-ESI-MS/MS are widely used.

UPLC is becoming increasingly popular due to its high sensitivity, high selectivity and lack of need for derivatisation. Sun et al. (60) used an isotopic displacement method coupled with UPLC–MS/MS in ESI positive ion mode and MRM mode to quantitatively analyze common contaminants, such as AA, HMF, and PhIP in fried meatballs. The method was rapid and sensitive, achieving high recovery and linearity (*R*^2^ > 0.9998) of all three analytes within 4.5 min. Edible insects are becoming increasingly popular in diet as they contain many micronutrients and high protein levels. However, acrylamide is generally produced in food processing. Therefore, its determination in insect food cannot be ignored. Simultaneous determination of furans and acrylamide in insect food by HPLC-QqQ-MS/MS was reported by González-Gómez et al. (61). This technique used acidified water for SLE followed by SPE using functionalized mesoporous structured silica as a solid-phase extraction sorbent. The recoveries of furan compounds and acrylamides were 70–101%, respectively, with an adequate precision (RSD < 9%) and good linearity (*R*^2^ ≥ 0.995). It can be seen that LC–MS plays an important role in the detection acrylamide in thermally processed foods, which is an important tool for food safety monitoring.

HRMS is particularly suited for the detection of low molecular weight amides, with simple extraction, no clean-up step and shorter chromatographic times than other MS techniques. Fernandes et al. (62) used LC-ESI-Orbitrap HRMS to identify and quantify acrylamide in specific food substrates of biscuits. The HRMS method is reliable, can accurately analyze acrylamide, and has low dependence on matrix composition. The method had good reproducibility, with a LOD of 3.55 μg/kg and a LOQ of 11.8 μg/kg. Troise et al. (64) used UHPLC-Orbitrap HRMS to determine acrylamide in biscuits, French fries, ground coffee and brewed coffee. The results were in perfect agreement with those obtained by the LC-MS/MS method. LOD was 2.65 ppb. LOQ was 5 ppb. UHPLC-Orbitrap HRMS is going to be a promising technique for determining acrylamide in food.

### Food additives

Food additives are added to preserve food’s flavor or improve its taste, appearance, and other qualities (67). Typical food additives include coloring, sweetening, preserving, antioxidant, and flavoring agents. Artificial foods have slowly taken over natural foods since they are cheaper. However, the widespread use of synthetic food additives can cause many problems related to misusing food additives, overdosing, and even toxicity. In addition, they pose potential risks to human health. Studies reveal that these artificial colors can cause allergy, asthma, damage DNA, cause hyperactivity, cancer, and human mutations. Therefore, screening food additives and quantifying their content is important since many unscrupulous companies use illegal additives for their benefit. MS is widely used in food analysis due to their multiple benefits. Table 5 summarizes the use of MS to analyze food additives.

**Table 5:** Applications of MS for analysis of food additives in foodstuffs.

Foodstuffs	MS	Analytes	Sample preparation	LODs	Reference
Cheese	MALDI-MS	Natamycin	Freezing at −80°C	–	(68)
Spices	UPLC-MS/MS	Rhodamine B	Grinding and homogenization Freezing at 4°C	0.1 μg/kg	(69)
211 Food and spice sample	UPLC-MS/MS	Auramine O	Homogenization	0.1 μg/kg	(70)
Functional food	ASAP-MS	Pharmaceutical drugs	–	10–20 μg/mL	(71)
Kimchi	HPLC-MS/MS	Synthetic food additives	–	0.00004–0.24 μg/mL	(72)

–, not mentioned.

Rhodamine B (RhB) is a prohibited food color additive. Many unscrupulous traders add RhB illegally to food products, especially spices. Numerous studies showed that RhB is carcinogenic, a neurotoxin, and a chronic toxicant for humans and animals. Wang et al. (69) used a sensitive UPLC-MS/MS method to monitor RhB in 292 different spices and reported that all samples were contaminated with RhB, suggesting that its exposure has a potential risk to consumers. Therefore, determining these illegal additives in food cannot be neglected. A synthetic color in food, AO is a non-approved food additive, but AO is still used for the color development of sour sprouts and chicken. Therefore, detecting this compound is vital, and a study was carried out to monitor the presence of AO in 211 samples of foods and spices using a UPLC-MS/MS method (70).

Improving in people’s living standards makes them more health-conscious, resulting in a growing demand for functional foods. Therefore, some people illegally add drugs to functional foods. When healthy people unknowingly consume these products, they suffer from side effects of decreased immunity, nutritional deficiencies, sickness, nausea, diarrhea, emesis, and kidney and liver damage. Wang et al. (71) proposed a new method for rapidly screening 42 widely used illegal additives in 6 functional food groups, based on an Atmospheric Solids Analytical Probe coupled with MS (ASAP-MS), without LC separation. The proposed method was used to analyze liquid or gaseous samples without pre-treatment. In addition, using a homemade library allowed rapid identification of suspect additives. The method was highly sensitive for samples in complex matrices, such as coffee samples. The ASAP-MS method was accurate, with the benefits of mobility, low cost, ease of operation, and automatic adjustment; thus, it can be widely used for rapid on-site detection of analytes in public security bureaus, especially grassroots police stations without corresponding equipment. Kim et al. (72) used LC-ESI-MS/MS to detect 7 food additives in kimchi with LODs of 0.00004–0.24 lg/mL, LOQs in the range of 0.00012–0.8 lg/mL, and the recoveries were 85.65–120.82%. Himmelreich et al. (68) determined preservative pseudomycin in cheese using MALDI-MS imaging (MALDI-MSI). MALDI-MSI can be used to detect a range of highly polar, non-volatile and thermally unstable samples. It is particularly promising for the detection of contaminants in food samples due to the reduced sample handling requirements and the ability to directly analyze untreated samples.

### PCBs and Dioxins

**Figure 3:**
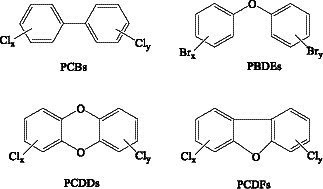
PCBs and dioxins structural formulae.

PCBs and Dioxins are toxic chemicals present in the environment and accumulate through the food chain, and both are highly toxic due to chlorine atoms (Figure 3). Table 6 summarizes the MS methods used to analyze PCBs, dioxins and contaminants in food.

**Table 6:** Applications of MS for analysis of PCBs and dioxins in foodstuffs.

Foodstuffs	MS	Analytes	Sample preparation	LODs	Reference
Cream and ice cream	GC-QqQ-MS/MS	NDL-PCBs	Homogenisation	0.04–0.16 ng/g	(73)
Catfish tissue	GC–MS/MS	19 PCB congeners	Homogenisation	–	(74)
Vegetables, beans, and grains	GC–MS	PCBs	Drying and grinding storing at 5°C	–	(75)
Chinese mitten crab food	GC-(APCI) MS/MS	PCBs, PCDD/Fs	Freeze-dried	PCBs: 0.021–0.150 pg./mL, PCDD/Fs: 0.051–0.237 pg./mL	(76)
Eggs, milk, fish, shellfish, pork, beef, and poultry	HRMS	PCDD/Fs, PCBs, PBDD/Fs, PBDEs	Homogenisation	–	(77)
Milk, fish oil, chicken, pork, fish, eggs, and a chicken compound feed	GC-ITMS/MS	PCDDs, PCDFs, dl-PCBs	Stored in the dark at 4°C Freezing at −20°C	PCDD/Fs: 0.1–0.93 pg./g, dl-PCBs: 0.1–0.89 pg./g	(78)
Milk	GC–MS/MS	PCBs	–	<0.6 pg./g	(79)

–, not mentioned.

PCBs are a mixture of 209 different compounds. Due to their extensive use, six kinds of NDL PCBs are regarded as proxies for PCBs in food and are highly dispersed in the surrounding environment (80). PCBs affect people through several routes, and food is the main source, including eggs, seafood (74), poultry, meat (81), milk, and dairy products. PCBs are neurotoxic, carcinogenic, reprotoxic, immunotoxic, hepatotoxic, and cardiovascular toxic to humans. Therefore, it is essential to determine the PCBs’ occurrence in foodstuffs. Liu et al. (82) reviewed the progress of PCB determination by GC–MS, GC–HRMS, and HPLC-MS/MS. Arshad et al. (75) monitored mean levels and toxic equivalents (TEQ) of PCBs by GC–MS in vegetables, pulses, and cereals in Khanewal and Multan, Pakistan. Although the mean concentrations and TEQs were safe, some vegetables and cereals still posed a moderate risk to human health due to their high consumption rates. Shahsavari et al. (73) used a modified QuEChERS extraction and GC-QqQ-MS/MS method to monitor the levels of the 6 NDL-PCBs in cream and ice cream, and the recoveries were 95.54–107.18%. Among these, the cream exhibited higher levels of NDL-PCBs than ice cream. Lu et al. (79) used d-SPE with GC–MS/MS to determine PCBs in milk and obtained LODs were < 0.6 pg./g; the recovery range was 82.8 to 106%. PCBs are present in food at extremely low levels and the diversity and complexity of food matrices make the determination of PCBs in food difficult. Therefore, it is important to develop more efficient, sensitive and accurate methods to detect and quantify PCBs. Dioxins are highly toxic and difficult to break down. They are usually present in food at ultra-trace levels. Consuming vegetables grown in PCDD/Fs contaminated soil can cause transient liver damage, peripheral nerve damage, and cancer. Therefore, their detection is is crucial to preventing health risks (83). Polybrominated dibenzo-p-dioxins and dibenzofurans (PBDD/Fs) are produced during combustion and are considered environmental contaminants as by-products of industrial chemicals. However, their presence in food samples has only been reported recently. The primary route of exposure is dietary intake, producing toxic reactions. In addition, PBDF was found in higher concentrations than PBDD (84). Currently, detection is usually by GC–MS, with HRGC–HRMS being the most accurate but time-consuming method. Li et al. (76) used GC-(APCI) MS/MS to determine PCBs (one to ten) and PCDD/Fs in the food web of Chinese hairy crabs. Results showed that GC-(APCI) MS/MS detection was related to HRGC–HRMS, and the PCB concentration was correlated. Major PCB congeners in the aquatic food web were dichlorobenzene and 3, 3-dichlorobiphenyl; however, other congeners, such as MoCB and DiCB, were also identified in large quantities. Malavia et al. (78) analyzed PCDDs, PCDFs, and dl-PCBs in food using GC-ITMS/MS and obtained consistent results between GC-ITMS/MS and GC-HRMS. Moreover, the GC-ITMS/MS method was more accurate and faster. Therefore, it was recommended to determine PCDDs/PCDFs and dl-PCBs in foodstuffs and animal feed samples. A combination of HRMS was used to accurately quantify PCDD/Fs, PCBs, PBBFs, and PBDEs in eggs, milk, fish, shellfish, pork, beef, and poultry (77). Analyses were accomplished using HRGC-HRMS with a Trace Series 1,310 GC with DFS. The sample contaminants (except for PCDD/Fs) were sprayed in undivided mode, and the injector operated in undivided surge mode. All four pollutants were present in the sample, with PCBs being the most prominent.

### PFCs and PPDs

Controlling the risks associated with changing mixtures of contaminants is one of the major challenges facing food safety today. Among the most prominent emerging food contaminants are PFCs and PPDs, which are of particular concern. PFCs are used in many applications, such as food packaging, nonstick pans, electronics, carpeting, fabrics, paints, adhesives, personal care products, and fire-fighting foam. The use and disposal of PFCs has resulted in the widespread distribution of these chemicals in the environment and their widespread presence in humans and wildlife. Bioaccumulation in fish has been shown to be a major source of PFCs in the diet. They are carcinogenic and are associated with hormonal disorders. In addition, they can accumulate and biomagnify through the food chain. PPDs is a synthetic compound and the newest pollutant, widely used to manufacture tires, belts, hoses, and cables. Due to the extensive use of these products in daily life, large quantities of PPDs and its breakdown products are released into the environment. Humans are exposed to PPDs and PPD-Q by inhalation, drinking water, eating, and skin contact. PPDs poses a potential risk to human health and causes angioneurotic edema, methemoglobinemia, acute tubular necrosis, and hepatotoxicity. Therefore, the determination of PFC and PPDs levels in food is essential for food safety. Table 7 summarises the MS methods used to analyse contaminated PFCS and PPD in food.

**Table 7:** Applications of MS for analysis of PFCs and PPDs in foodstuffs.

Foodstuffs	MS	Analytes	Sample preparation	LODs	Reference
Milk	MIP-PR-DFE-LC–MS/MS	PFOA and PFOS	–	0.006–0.022 ng/mL	(85)
Chicken eggs, quail egg	LC–MS/MS	PFOA and PFOS	Freezing at −20°C	–	(86)
Honey	Micro-UHPLC–MS/MS	PFOA and PFOS	–	PFOA: 0.016 μg/kg PFOS: 0.040 μg/kg	(87)
Fish, meat, offal, egg, cracker, chips, cake, chocolate, vegetable, milk, and juice	LC–MS/MS	PFOA and PFOS	Rinsed in distilled water and blotted dry.	PFOA: 0.038 ng/g PFOS: 0.002 ng/g	(88)
Rainbow trout, brook trout, Arctic char, and white sturgeon	UHPLC-Q-Exactive HF-Q-Orbitrap HRMS	6PPD-Q	–	–	(89)
Salmonid Species	LC–MS/MS	6PPD-Q	–	–	(90)
Fish and honey	HPLC-MS/MS	6PPD and 6PPD-Q	Fish homogenization Freezing at −20°C Honey samples were kept at 25°C	6PPDQ: 0.0003 mg/kg 6PPD in fish 0.00025 mg/kg, 6PPD in honey 0.0003 mg/kg	(91)
Lettuce	HRMS	6PPD and 6PPD-Q	Freezing at −20°C	–	(92)

–, not mentioned.

**Figure 4:**
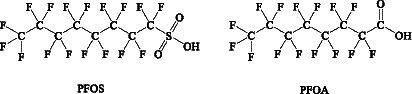
Chemical structures of PFOS fand PFOA.

PFCs are a large group of man-made organic chemicals. The two most hotly debated PFCs are perfluorooctane sulfonic acid (PFOS) and perfluorooctanoic acid (PFOA). The PFOA and PFOS structures are shown in Figure 4. They have thermal and chemical inertness, high surface activity, and relatively low surface energy. Therefore, they are widely used in public and industry. Several PFOAs have been identified as persistent, bioaccumulating, and toxic. Therefore, it is important to determine the PFOA and PFOS levels in food. Their highest levels are known to occur in the Arctic. Kantiani et al. (93) reviewed industrial organic contaminants PFCs in food. They presented the major techniques used to detect and quantify them in food. Marine bioaccumulation is a major dietary source of PFCs. A recent review described several new PFAS methods published in the past 2 years, using ion mobility spectrometry (IMS) and MS (94).

Surma et al. (87) applied d-SPE and micro-UHPLC–MS/MS to determine PFOA and PFOS in honey samples, using ENV as sorbent and acetonitrile as an extraction solvent. Obtained results showed that the best recoveries were 84 and 87% for PFOA and PFOS, respectively. Ren et al. (85) recently used a novel MIP-PR-dispersion filter extraction (DFE) coupled with LC–MS/MS to determine PFOA and PFOS in milk. Compared with previously established methods, the proposed MIP-PR-DFE method combined the strengths of d-SPE and SPE with good cleaning performance and recovery regarding speed, selectivity, and cost-effectiveness. DFE was applied for the first time with LC–MS/MS to analyze PFOA and PFOS in milk, providing a new approach for efficient pollutant detection in food chemistry. The analysis results showed excellent recoveries (94.7–109%) and precision (RSD ≤9.5%). Sungur et al. (88) used LC–MS/MS to monitor PFOA and PFOS in 123 foods and beverages, such as fish, meat, offal, eggs, biscuits, French fries, cakes, chocolate, vegetables, milk, and fruit juices and described that fish was the main source of PFOS intake, while meat and offal were main sources of PFOA intake. Later, Tahziz et al. (86) determined PFOS and PFOA in egg yolk samples using LC–MS/MS. PFOS was quantified with concentrations ranging between 0.5 and 1.01 ng/g. The developed technique was economical, labor-saving, and sensitive. In conclusion, LC–MS is a good tool for the detection of PFOA and PFOS in food.

**Figure 5:**
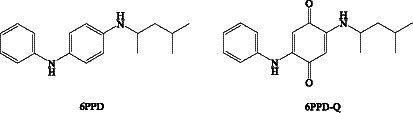
Chemical structures of 6PPD and 6PPD-Q.

In China, the annual production of 6PPD is the highest of the PPD antioxidants. The rubber antioxidant (6PPD) and its ozonation product (6PPD-Q) are widely found in air (95), dust (96), and water (97, 98). Tian et al. (99) reported that 6PPD-Q causes acute pre-spawning mortality of silver salmon in freshwater streams of the Pacific Northwest, a phenomenon known as ‘urban runoff mortality syndrome’. This groundbreaking study generated great interest and concern. 6PPD-Q is a greater toxicant than its parent compound 6PPD, and contact with tire rubber-derived contaminants 6PPD-Q can induce *in vitro* mitochondrial dysfunction (90). The 6PPD and 6PPD-Q structures are shown in Figure 5. These compounds are widely found in vegetables, animal foods, or seafood.

The effects of 6PPD-Q on 4 commercially, culturally, and ecologically important fish species, including rainbow trout, brook trout, Arctic charr, and white sturgeon, were investigated by Brinkmann et al. (89) using UHPLC-Q-Exactive HF-Q-Orbitrap HRMS. This work demonstrated the acute toxicity of 6PPD-Q at environmentally relevant concentrations to other commercially, culturally, and ecologically important fish species. Salmonids are taxa of great cultural, ecological, and market value worldwide. Hiki et al. (100) conducted a 6PPD-Q 96-h acute toxicity test using LC–MS/MS on three salmonids, showing lethal toxicity to white-spotted salmon. Ji et al. (91) developed a modified QuEChERS method combined with HPLC–MS/MS to study the 6PPD and 6PPD-Q levels in fish and honey. Both these compounds were detected in blackfish, sea bass, and horse mackerel, while none were in honey samples. The linearity, recovery, and matrix effects were satisfactory, ranging from 70.4 to 95.6%, with excellent reproducibility (RSD < 8.4%). This work demonstrated the presence of 6PPD and 6PPD-Q in fishery samples for the first time, suggesting that they could enter the human body by eating polluted fish. To source and quantify the TWPs-derived compounds in edible plants. Castan et al. (92) first incubated lettuce plants in a TWPs-derived hydroponic solution. Their leaves, roots, and nutrients were collected for processing, and QMS determined concentrations. In addition, the compounds in plant leaves were identified by high-resolution Orbitrap MS. This work indicated that TWP could be a persistent source of TWP-derived compounds in edible plants, with TWP metabolites accumulating in lettuce leaves. In addition, 6PPD was readily absorbed by lettuce. 6PPD has been detected in environmental matrices, including water, soil, and particulates, so lettuce safety should also be a cause of concern. In summary, MS is an useful tool for the determination of new contaminants and LC–MS is very beneficial for the screening of unknown pollutants in food.

## Conclusions and future perspectives

This work completely reviewed MS methods in analysis of harmful contaminants in food. The combination of MS with modern chromatographic and other separation tools offers higher resolution and precision than conventional MS, enabling isomeric and conformational analogs’ identifications. Compared to conventional chromatographic approaches, LC–MS and GC–MS allow more rapid and sensitive detection of toxic and hazardous substances in food, which can effectively solve food safety emergencies. Moreover, MS can identify unknown compounds from complex background matrices and consequently has been fully implemented in routine analytical and research laboratories. The capabilities of MS-based proteomics are expanding with recent advances in MS technologies. HRMS methods provide rapid and comprehensive information on food contamination via targeted and non-targeted analysis, which is a breakthrough. However, the standardization of untargeted analysis still needs much work. Furthermore, there are emerging food safety issues, like the misuse of antibiotics, the application of toxic nanomaterials (e.g., food packaging materials) and the occurrence of unreported contaminants. Hence, development of more rapid, sensitive and accurate MS approaches in food analysis is still needed.

## Author contributions

QS wrote the original draft of the manuscript. QS, YD, XW, XZ, and SH conducted the searching processes. WZ and DY reviewed, edited, and supervised the manuscript and done the funding acquisition. All authors contributed to the article and approved the submitted version.

## Funding

This work was supported by the National Natural Science Foundation of China (22206172, 22076174).

## Conflict of interest

The authors declare that the research was conducted in the absence of any commercial or financial relationships that could be construed as a potential conflict of interest.

## Publisher’s note

All claims expressed in this article are solely those of the authors and do not necessarily represent those of their affiliated organizations, or those of the publisher, the editors and the reviewers. Any product that may be evaluated in this article, or claim that may be made by its manufacturer, is not guaranteed or endorsed by the publisher.
